# Chirality on Amorphous High-*T*_g_ Polymeric Nanofilms: Optical Activity Amplification by Thermal Annealing

**DOI:** 10.3390/nano7080208

**Published:** 2017-08-01

**Authors:** Tiziana Benelli, Massimiliano Lanzi, Laura Mazzocchetti, Loris Giorgini

**Affiliations:** 1 Dipartimento di Chimica Industriale Toso Montanari and INSTM UdR-Bologna, University of Bologna, Viale Risorgimento 4, 40136 Bologna, Italy; tiziana.benelli@unibo.it (T.B.); massimiliano.lanzi@unibo.it (M.L.); laura.mazzocchetti@unibo.it (L.M.); 2 Interdepartmental Center for Industrial Research on Advanced Applications in Mechanical Engineering and Materials Technology (CIRI-MAM), University of Bologna, Viale Risorgimento 2, 40136 Bologna, Italy

**Keywords:** azobenzene containing-polymers, conformational chirality, chiral polymers, circular dichroism, chiral amplification

## Abstract

The chiroptical properties of amorphous chiral polymers functionalized with conjugated *trans*-azoaromatic chromophore linked to the backbone through a chiral cyclic pyrrolidine moiety of one single configuration at the solid state, as thin films, were investigated. For the first time nanometric thin films of amorphous polymers (not liquid crystals) showed a remarkable chiral amplification upon thermal treatment at a temperature close to their *T*_g_. The side-chain azobenzene chromophores rearrangement driven by the enhanced chain mobility seems to favor the formation of nanodomains of conformationally ordered macromolecular chains with one prevailing helical handedness whose optical activity depends on the configuration of the intrinsic chirality of the monomeric units and which as a result are stable at room temperature for a long time.

## 1. Introduction

The Azobenzene derivatives are well known as photoresponsive materials: indeed, when subjected to UV light irradiation, the thermodynamically stable *trans*-isomer can be converted to the *cis*-one, which can back isomerize to the more stable *trans*-isomer either by photoexcitation or upon heating in the dark. The isomerization process appears, for this class of materials, highly effective and easily tunable, even when azobenzene moieties are bonded to polymeric structures [1,2,3,4,5,6,7]; this behavior makes them potential candidates for applications in a number of technological fields, such as devices for the optical storage of information [8,9], waveguides, holographic memories [10,11], nonlinear optical materials [12], optical input/electrical output memory devices [13], chemical photoreceptors [14,15], photoconductive and photorefractive materials [16,17] and, in general, as materials which, upon irradiation with light of appropriate intensity and frequency, exhibit photoresponsive properties [18,19,20,21,22,23,24].

In addition, when the *trans*-azoaromatic moieties belong to a polymer’s side chain, if the chromophores are linked to the polymeric backbone through chiral groups of one prevailing configuration [25], the whole macromolecule is brought to assume a conformational dissymmetry which can be revealed by Circular Dichroism (CD) measurements [17,26,27]. 

It is worth noting that the induction of helical handedness in polymers [28] can be exploited in the design of optical devices or for data storage, other than because of its relevance to chiral amplification [5,29,30,31]. Several studies were reported for different systems both in solution [32] and in the solid state [31,33,34]. For example Green and co-workers reported the possibility for inducing a helical conformation with a prevailing screw sense in polyisocyanates in dilute solution by functionalization of the macromolecules with chiral pendant groups having a small enantiomeric ratio [5,35] or by photoresolution [36].

Furthermore, recently Guerra et al., investigated the chiroptical response of racemic polymers in the solid state which are able to form co-crystalline phases with chiral low-molecular-weight guests [33]. This phenomenon can be produced by molecular and supramolecular mechanisms which are of configurational and conformational origin.

In this context, Liquid Crystal (LC) polymers represent a peculiar class of materials owing to their ability, when they are assembled as thick films (micron), to show chiral amplification due to the long-range positional and orientational organization of mesogenic groups that produces a chiral superstructure [37,38].

Particular attention was given to the amplification of chiroptical properties of polymeric films by photoirradiation. For example Nikolova and co-workers [39,40], Natansohn and co-workers [41], and Sourisseau and co-workers [42] reported the induction of optical activity on achiral azobenzene-containing polymers by irradiation with one-handed circularly polarized (CP) light. In those achiral systems, however, the chromophores require a preliminary alignment along a preferential direction, obtained by irradiation with linearly polarized (LP) light or by a liquid crystalline phase transition. In other words, this phenomenon is possible only if specific ordered chromophoric nanodomains are pre-formed.

By contrast, the photomodulation of the chiroptical properties of amorphous and intrinsically chiral azobenzene-containing materials does not require such preliminary treatments [43,44,45].

It is worth noting that in literature are present also examples of thermal modulation for data-storage applications. Among the others, Fujiki et al. reported versatile solid-film polysilanes exhibiting chiroptical switching and/or chiroptical memories with write-once read-many (WORM) and re-writable (RW) modes based on helix-helix transitions driven by the temperature [46].

In this context, we already reported the investigation of optically active photochromic homopolymeric derivatives with pendant *trans*-azoaromatic moieties with permanent dipole moment, connected to the backbone via a chiral pyrrolidinyl ring of one prevailing absolute configuration [26,47,48,49]. The simultaneous presence of these functional groups allows the polymers to contemporarily display both the properties typical of dissymmetric systems (optical activity, exciton splitting of chirooptical absorptions), and those characterizing photochromic materials (photorefractivity, photoresponsiveness, nonlinear optical properties). In particular, the strong optical activity displayed by these materials, both in solution and as solid thin films, indicates that the macromolecules are able to assume highly homogeneous conformations with a prevailing chirality sense owing to the instauration of electrostatic interactions between the *trans*-azoaromatic moieties which are disposed according to a chiral geometry. Thus, their CD spectra show exciton splitting of the dichroic bands, whose extent depends on the extent of the interactions and therefore on the overall amount of chiral conformations assumed by the macromolecules [47,48,49].

Further, studies demonstrated that the chirality manifestations of these materials strongly depend on their molecular weight [49,50] and that also short chain sections significantly contribute to the overall optical activity, as previously discussed in a study focusing on dimeric derivatives which represent the smallest section of polymer where interchromophore interactions can set in [51]. 

**Figure 1:**
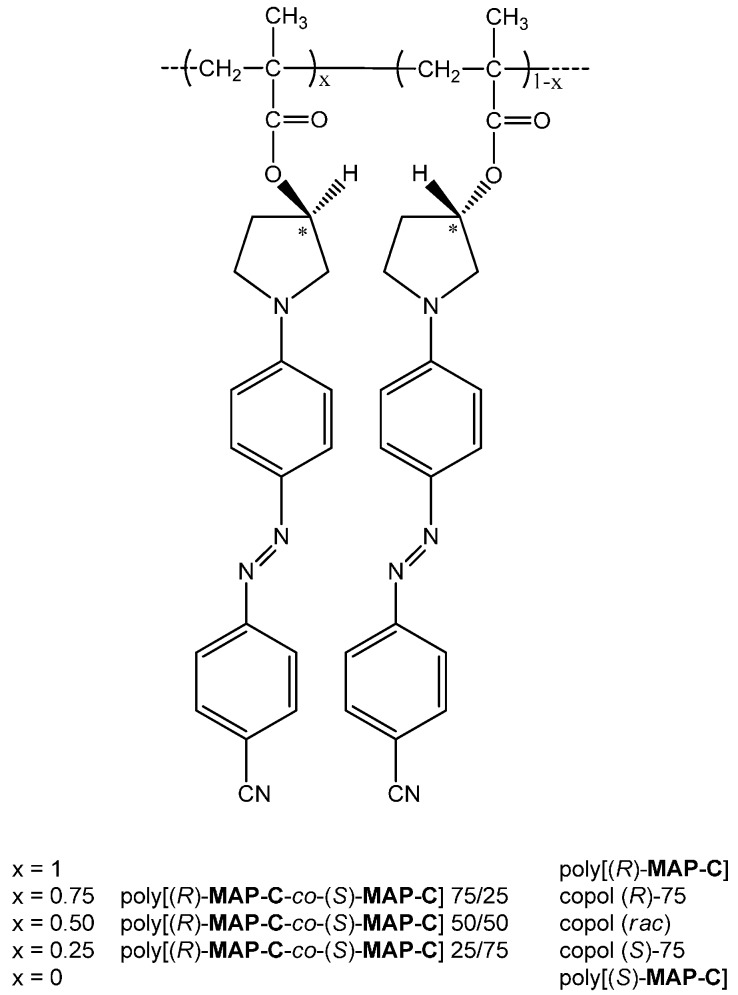
Chemical structure of the investigated azobenzene derivatives.

With the aim of studying the effect of temperature on the chiroptical properties of this class of materials originated by macromolecular conformational helix order, amorphous thin films of the two enantiomeric homopolymers poly[(*S*)-3-methacryloyloxy-1-(4′-cyano-4-azobenzene)pyrrolidine] {poly[(*S*)-**MAP-C**]} [47] and poly[(*R*)-3-methacryloyloxy-1-(4′-cyano-4-azobenzene)pyrrolidine] {poly[(*R*)-**MAP-C**]} [48] were characterized and their thermally induced chiral organization was compared to the starting one and to that of the corresponding random copolymers of the two enantiomeric monomers (*R*)-**MAP-C** and (*S*)-**MAP-C** (Figure 1) with different molar composition. Particular attention was given to the study of the circular dichroism spectra of the materials heating at different times and temperatures.

## 2. Results

Amorphous thin films of previously synthesized homo- and co-polymers [47,48] were prepared by spin coating of the polymer solutions over fused silica. The thickness of each film was measured by a profilometer and was found to be in the range 150–350 nm. Their optical isotropy was assessed with a cross-polarized optical microscope.

### 2.1. UV-Vis Absorption and Chiroptical Properties in the Solid State

**Figure 2:**
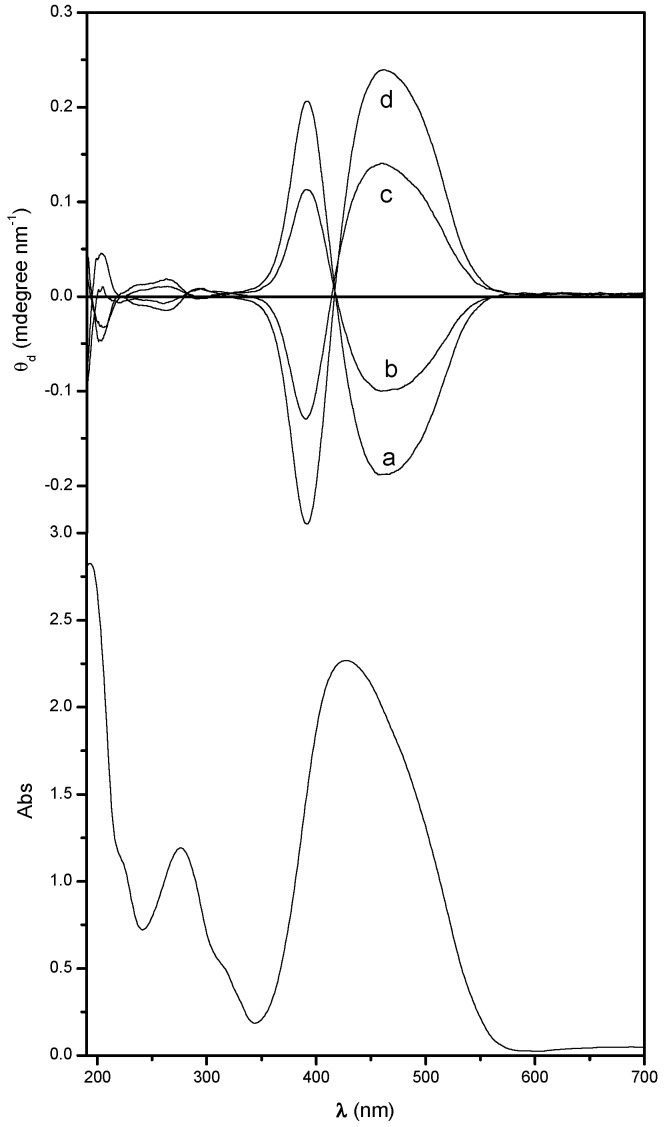
Bottom: UV-Vis spectrum of a 270 nm thick film of poly[(*S*)-**MAP-C**]. Up: CD spectra (normalized by the film thickness) of poly[(*R*)-**MAP-C**] (a); copol (*R*)-75 (b); copol (*S*)-75 (c) and poly[(*S*)-**MAP-C**] (d) at the solid state.

The UV-Vis absorption data of the studied polymers as amorphous thin films are given in Table 1. As expected, the absorption spectra of all these azobenzene derivatives are very similar to each other, showing, in the spectral region 700–190 nm, two absorption bands (Figure 2 bottom): the more intense one is centered at about 425 nm and is due to the *n*–π*, π–π* and internal charge transfer electronic transitions of the conjugated azoaromatic chromophore; the other one, positioned around 275 nm, is related to the π–π* electronic transition of the aromatic ring [47,48]. 

The registered spectra appear quite similar to the previously reported ones for the same systems in DMA dilute solutions [47,48] even though the first absorption band results are blue-shifted by about 20–25 nm (Table 1). In agreement with several reports [52,53,54], such a behavior suggests the formation of H-type chromophoric aggregates (which consist of an intramolecular parallel arrangement of the azobenzene electric dipoles) forced by the structural constraints of the macromolecules [43,47] as a result of solvent removal. The blue shift is even more evident if we consider the spectrum of the monomeric model compound (*S*)-3-pivaloyloxy-1-(4′-cyano-4-azobenzene) pyrrolidine [(*S*)-**PAP-C**] (Table 1) which lacks any structural restriction. Thus, the further shift observed upon passing from solution to solid state can be ascribed to an increase of the H-type chromophoric aggregates as a consequence of the removal of the solvent and the collapse of the polymer chains to form the film. 

The CD spectra of the amorphous polymers in the solid state are also very similar to those in dilute solutions [47,48] (Table 2 and Figure 2 up). The two enantiomeric homopolymers, indeed, show an exciton splitting of opposite sign originated by cooperative interactions between side-chain azochromophores disposed in a mutual chiral geometry of one prevailing and opposite handedness at the wavelength of the maximum of the visible absorption band [55,56]. Such a behavior confirms that the two homopolymers assume, also in the solid state, at least for chain sections, enantiomeric conformations of one prevailing screw sense related to the absolute configuration of the starting monomer.

By decreasing the relative content of repeating units of one prevailing configuration, the intensity of the dichroic couplet is reduced, as demonstrated by the CD spectra of copol (*R*)-75 and (*S*)-75 (Figure 2 up); as expected, no dichroic signals are present in the spectrum of the racemic one [copol (*rac*)].

The same trend was previously shown by these materials in diluted DMA solution [48], thus demonstrating that the dipolar interactions between chiral groups of identical absolute configuration, which are responsible for this phenomenon, are maintained (freezing) also in the solid state.

### 2.2. Annealing

The amorphous thin films of the two enantiomeric homopolymers and the copol (*rac*) were heated in air for a given amount of time at constant temperature, then rapidly cooled at room temperature and their CD and UV-vis spectra recorded. Each sample was submitted to several such thermal cycles, applying progressively higher temperatures every cycle, as many times as required for two subsequent spectra to not show any further change. As expected, no variations on CD and UV-vis spectra were detected by annealing the samples for 60 min at temperatures far from their glass transition temperature (*T*_g_ about 185–192 °C determined on the polymers as a powder) [47,48], e.g., lower than 170 °C.

**Figure 3:**
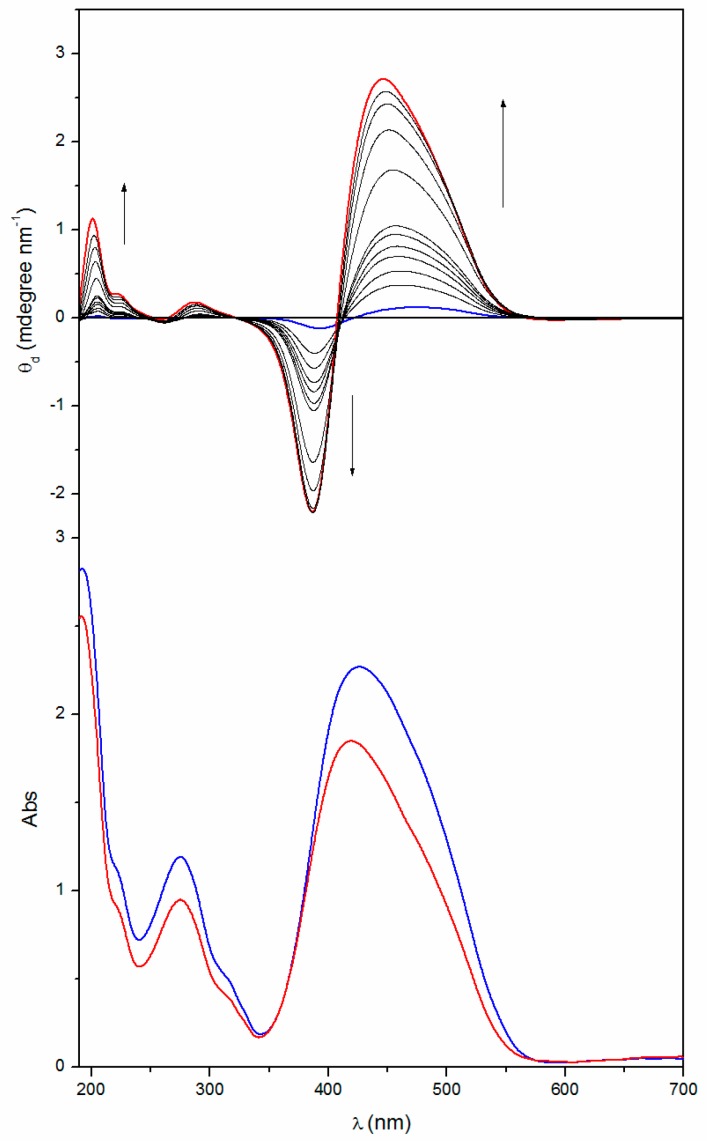
CD (up) and UV-Vis (bottom) spectra of a 270 nm thick film of poly[(*S*)-**MAP-C**] before (—) and after annealing at 200 °C for 5, 10, 15, 20, 25, 30, 40, 50, 60, 75 (—) and 100 (—) min. CD spectra are normalized for the film thickness.

**Figure 4:**
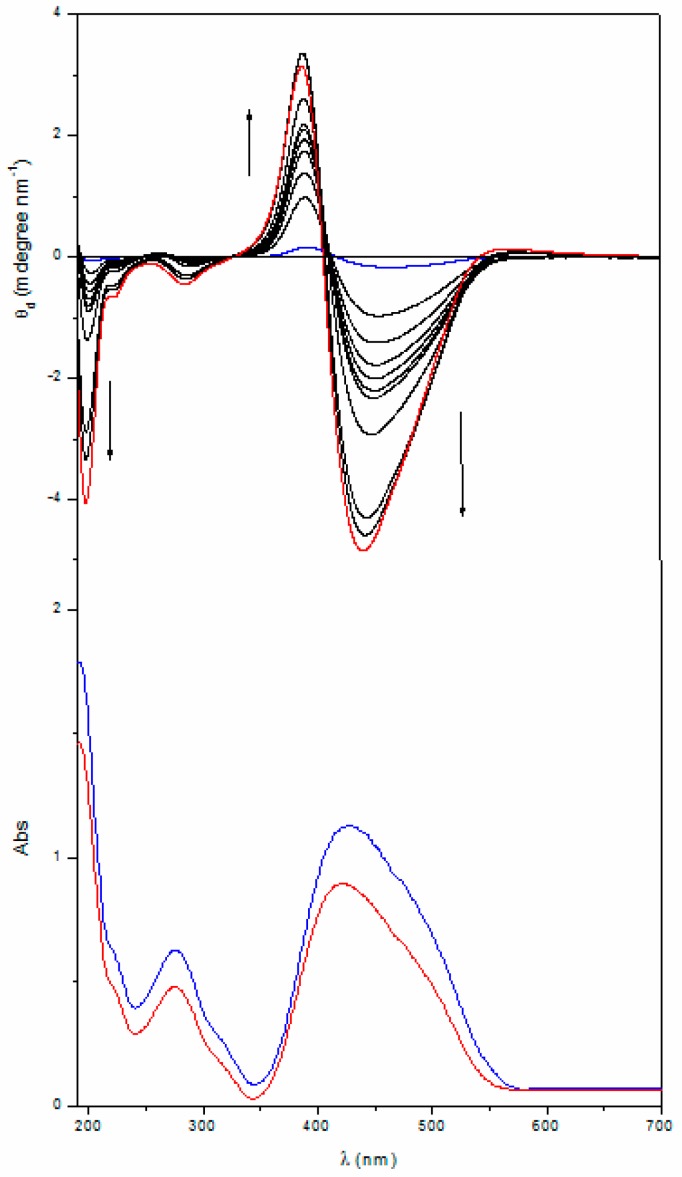
CD (up) and UV-vis (bottom) spectra of a 145 nm thick film of poly[(*R*)-**MAP-C**] before (—) and after annealing at 200 °C for 5, 10, 15, 20, 25, 30, 40, 50, 60 (—) and 75 (—) min. CD spectra are normalized for the film thickness.

At a higher temperature (200 °C) a significant change of the UV-vis and CD bands of the homopolymers is observed; such a behavior can be ascribed to some kind of thermal transition experienced by the thin film samples. As an example, Figure 3 and Figure 4 display the UV-vis and CD spectra of the thin films of the two homopolymers recorded at different times upon annealing at 200 °C, just above their glass transition temperatures.

It is evident that, by annealing at a temperature close to the *T*_g_, the absolute intensity of all the CD bands progressively and considerably enhances, and saturates at a value that is at least 20 times larger than that of the native films. Although their resulting shape and position are unchanged, the cross-over point of the exciton couplet in the Visible region progressively moves towards shorter wavelengths (about 15–20 nm). As expected, no changes were registered on the CD spectra of the annealed copol (*rac*) which remains silent.

It is worth noting that remarkable modification of the UV-vis absorption spectra of the polymer films were also recorded (Figure 3 and Figure 4 bottom). Upon annealing, indeed, the visible absorption band shows a significant intensity reduction together with a further 8–10 nm blue shift of the band maximum with respect to the native film. The shift of the visible absorption band to shorter wavelengths, observed after prolonged thermal treatments of the film, suggests an enhancement of the degree of the chromophore aggregation (H-type aggregation) [43,44,45]. This reorganization of the dipolar interactions between chromophores in the side chain could also explain the dramatic changes of the CD spectra reported above.

It is worth noting that all the annealed films, as well as the related native ones, were optically isotropic (no LC phases were detected, thus no LC chiral suprastructures with macrodomains conformationally ordered are present).

**Figure 5:**
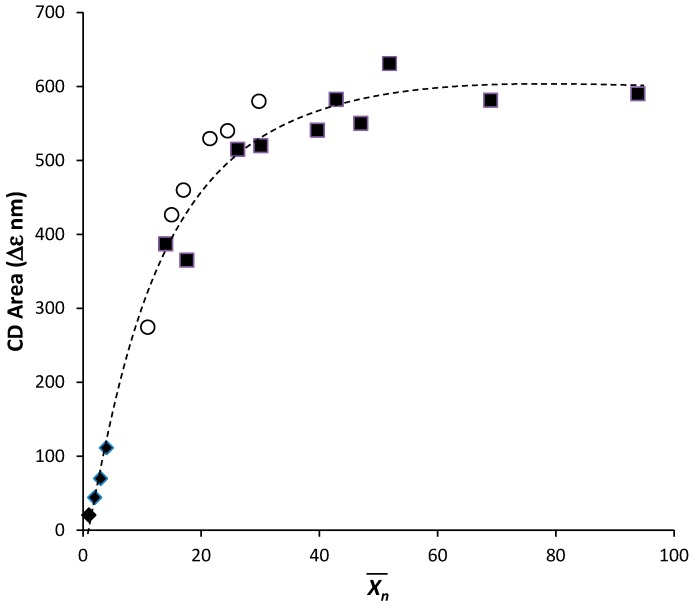
Evolution of the amplitude of the CD exciton couplet versus the average number polymerization degree relative to the oligomeric derivatives (♦) [50] and polymeric samples of poly[(*S*)-3-methacryloyloxy-1-(4-azobenzene)pyrrolidine] [poly[(*S*)-**MAP**] obtained by different free and living radical polymerization methods (■) [50] (○) [49].

Previous studies demonstrated that the optical activity of these materials is not related to the presence of a predominant configuration of the stereogenic centers in the backbone but is essentially of conformational origin [46]. Moreover, investigations on analogous azobenzene chiral methacrylic polymers [49,50] highlighted that the CD bands are strongly dependent on the average polymerization degree of the macromolecules, tending to attain the highest and constant amplitude at *X_n_* around 20–25 (Figure 5), and short chain sections are already able to contribute to the overall optical activity.

In particular, it was demonstrated that the interactions between adjacent side-chain chromophores having conformational dissymmetry of one prevailing screw sense for short chain sections already constitute a relevant contribution to the overall chirality manifestations of the material, in accordance with the results previously achieved by investigating the spectroscopic and chiroptical properties of a similar dimeric derivative containing two photochromic chiral moieties [51], the smallest section of polymer where interchromophore interactions can be present.

**Figure 6:**
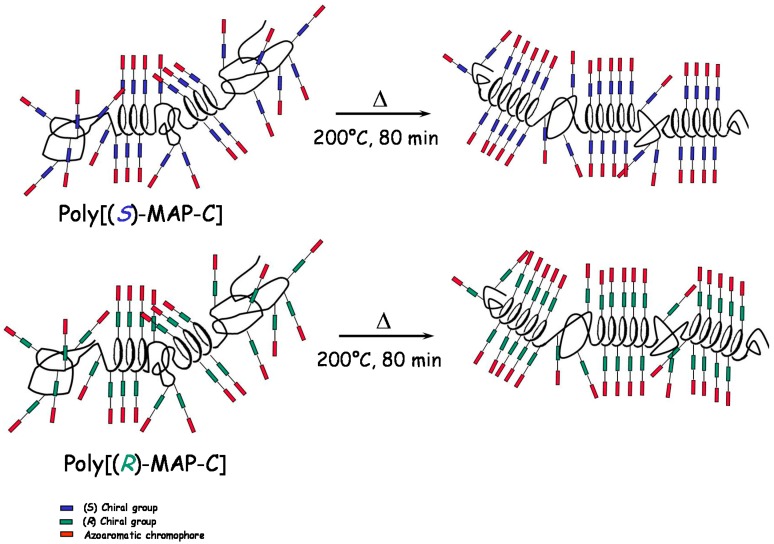
Idealized pictures of thermoinduced conformational chiral order of the macromolecules.

Taking into account that the films are subjected to a temperature slightly above their glass transition, a rearrangement of the azobenzene chromophores can be envisaged, driven by the enhanced chain mobility allowing for a more thermodynamically favored chiral organization of the same chirality sign of the starting one. The strong reorganization of the dipolar interactions in the solid state subsequently brings in an increase of the macromolecular suprastructure and/or of the chain sections characterized by a helical structure that could explain the observed chiral amplification, as previously described by Saxena et al., for polysilane film [57] and by Zou et al., for azobenzene-substituted polydiacetylenes [58]. The enhanced optical activity of the amorphous thin films upon annealing could thus be ascribed to the formation of nanodomains of chromophores aggregated (H–type) in a chiral conformation with a prevailing helicity whose chirality is driven by the intrinsic optical activity of the macromolecules and which are frozen in the solid state as idealized in Figure 6.

**Figure 7:**
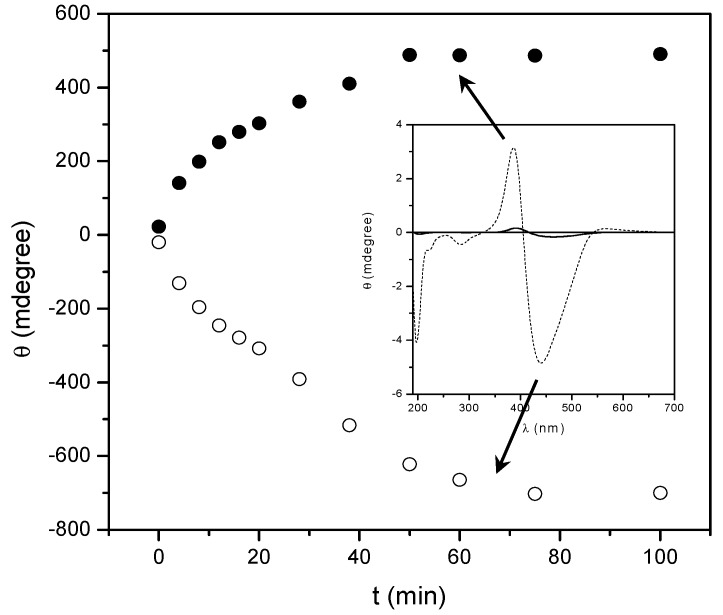
Evolution of the ellipticity at 385 (●) and 450 nm (○) versus the annealing time relative to poly[(*S*)-**MAP-C**] amorphous thin film heating at 200 °C.

**Figure 8:**
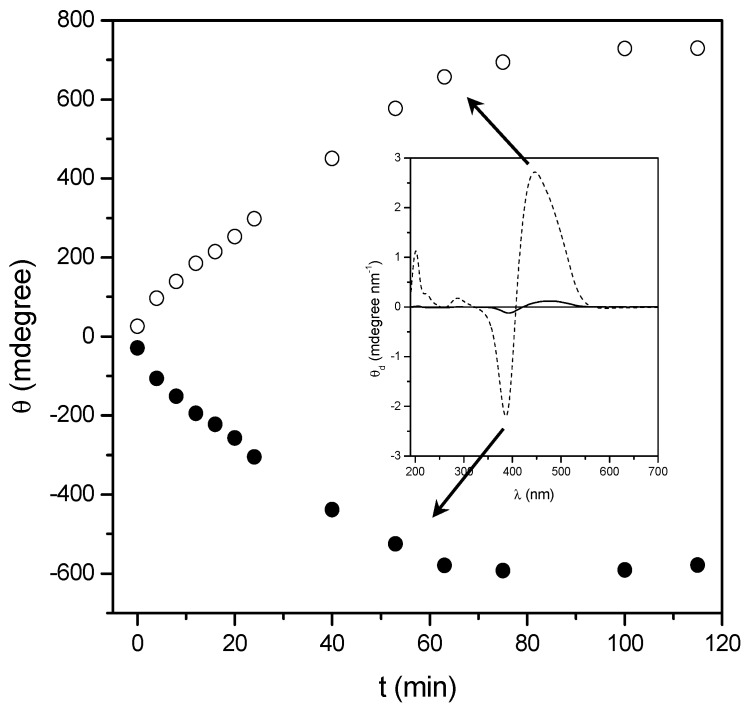
Evolution of the ellipticity at 440 (○) and 385 nm (●) versus the annealing time relative to poly[(*R*)-**MAP-C**] amorphous thin film heating at 200 °C.

To evaluate the evolution of chiral conformations assumed by the macromolecules in the solid state during annealing, we report in Figure 7 and Figure 8 the ellipticity registered at a wavelength close to the maxima of the two dichroic bands constituting the excitonic couplet, as a function of heating time. 

The CD signals tends to increase their amplitude progressively (with a quasilinear behavior) as a function of annealing time up to 80 min, where the amplitude reaches an almost constant value. Stemming from the knowledge that the chiroptical properties of interacting chromophores strongly depend on their dihedral angle and relative distance (R) (by a factor of about 1/R^6^), as stated by the model of electrostatic dipolar interchromophore interactions adopted to describe the CD spectra [56,59,60], this fact can be invoked to explain the previously discussed behaviour. In fact, by increasing the number of repeating units included within an ordered section, R also increases and consequently the interactions of a given chromophore progressively decay from the first neighbouring azobenzene, to the following one, and so forth, with convergence of the intensity of the CD signals to one asymptotic value.

An evaluation of the persistence with time of the conformational arrangement assumed by the macromolecules after annealing at a temperature around the *T*_g_ was made by keeping the film at room temperature for almost 6 months. After this time, the appearance of the UV and CD spectra is the same as that shown by annealing films for 75 min, thus suggesting that the above-mentioned thermal transition takes place rapidly at the *T*_g_ and does not produce further structural changes at room temperature. In conclusion, the thermal stability displayed by these chiral materials suggests that the resulting thermally induced chiroptical properties are stable at room temperature for a long period of time.

Though some light has still to be shed on the mechanism underlying the chiral amplification shown by these materials after thermal treatment, to the best of the authors’ knowledge this is the first time this effect has been reported for polymers which are not liquid crystals.

## 3. Materials and Methods

### 3.1. Physico-Chemical Measurements

Amorphous thin films of the studied materials were prepared by spin-coating a solution of the polymer in 1-methyl-2-pyrrolidinone/tetrahydrofuran (NMP/THF) over fused silica. The films were then dried by heating above 80 °C under vacuum for 12 h and stored in the dark. The films’ thickness, measured by a Tencor P-10 profilometer (KLA Tencor, Milpitas, CA, USA), was in the range 150–350 nm, depending on the procedure conditions. The native films were optically isotropic by inspection with a Zeiss Axioscope2 (Zeiss, Jena, Germany) polarising microscope through crossed polarizers fitted with a Linkam THMS 600 (Linkam Scientic, Surrey, UK) hot stage. 

UV-Vis absorption spectra of the thin films were recorded in the 700–190 nm spectral region with a Perkin-Elmer Lambda 19 spectrophotometer (Perkin-Elmer, Waltham, MA, USA).

CD spectra of the thin films were recorded on a Jasco 810 A dichrograph (Jasco, Esaton, MD, USA) and the data were normalized by the film thickness. The samples were submitted to heating in air at 200 °C (annealing) for a known amount of time, then rapidly cooled at room temperature and their CD and UV-vis spectra recorded. This cycle was repeated on the same sample at progressively longer times as long as the CD spectra did not show any further change.

### 3.2. Materials

The methacrylic homopolymers poly[(*S*)-3-methacryloyloxy-1-(4′-cyano-4-azobenzene) pyrrolidine] {poly[(*S*)-**MAP-C**]} and poly[(*R*)-3-methacryloyloxy-1-(4′-cyano-4-azobenzene) pyrrolidine] {poly[(*R*)-**MAP-C**]} and related copolymers poly[(*R*)-**MAP-C**-*co*-(*S*)-**MAP-C**] 75/25, 50/50 and 25/75 were synthesized as previously reported [47,48]. Characterization data concerning these materials are given in Table 3.

The low molecular weight structural model (*S*)-**PAP-C** [30] was crystallized from Abs. EtOH before use. 

Tetrahydrofuran (THF) and 1-methyl-2-pyrrolidinone (NMP) were purified, dried and stored under nitrogen over molecular sieves (4 Å). 

All other reagents and solvents (Sigma-Aldrich SRL, Milano, Italy) were used as received without further purification.

## 4. Conclusions

In this paper, we reported on the effects related to the heating of amorphous thin films of chiral azobenzene containing materials close to their glass transition temperature.

In particular, we studied the properties of two enantiomeric homopolymers and related copolymers containing different amounts of the two enantiomeric monomers. The CD spectra of the whole series confirm that these materials in the solid state maintain a predominantly helical structure with a well-defined sense related to the absolute configuration of the starting monomer also in the solid state. For the native amorphous films, the intensity of the CD signals enhances with the increase of the average length of the chain sections having one prevailing enantiomeric composition, as a consequence of dipolar interactions between chiral groups of identical absolute configuration. As expected, no dichroic signals are present in the spectrum of the polymer resulting from racemic monomers.

By heating the thin films of the two homopolymers close to their glass transition temperatures (200 °C), we observed a large and stereospecific increase of the CD properties of these chiral amorphous polymers up to final saturation.

Considering what was highlighted by previous studies on the relevant contribution of short chain sections of chromophores interactions on the overall optical activity of these materials, the reported behavior suggests an azobenzene rearrangement driven by the enhanced chain mobility at a temperature close to *T*_g_ which leads to a more thermodynamically favoured chiral organization in a nanodomain of the same sign as the starting one. 

## Figures and Tables

**Table 1:** UV-Vis spectra of azobenzene derivatives.

Sample	λ_max_^a^ (ε_max_10^−3^) ^b^	λ_max_^a^ (ε_max_10^−3^) ^b^
Poly[(*R*)-**MAP-C**] DMA ^c^	450 (33.3)	276 (13.3)
Poly[(*R*)-**MAP-C**] film	424	276
copol (*R*)-75 DMA ^c^	449 (33.6)	273 (14.4)
copol (*R*)-75 film	425	276
copol (*rac*) DMA ^c^	448 (35.0)	277 (13.7)
copol (*rac*) film	425	276
copol (*S*)-75 DMA ^c^	448 (34.8)	276 (14.0)
copol (*S*)-75 film	424	276
Poly[(*S*)-**MAP-C**] DMA ^d^	447 (32.4)	277 (12.4)
Poly[(*S*)-**MAP-C**] film	426	275
(*S*)-**PAP-C** DMA ^d^	458 (35.4)	277 (12.5)

^a^ Wavelength of maximum absorbance, expressed in nm. ^b^ ε_max_ in DMA solution expressed in L mol^−1^ cm^−1^ and calculated for one single chromophore. ^c^ Ref. [48]. ^d^ Ref. [47].

**Table 2:** CD spectra of thin films of the studied azobenzene derivatives before and after annealing at 200 °C for 100 min.

Sample	1st Absorption Band					2nd Absorption Band				
	λ_1_^a^	θ_d1_^b^	λ_0_^c^	λ_2_^a^	θ_d2_^b^	λ_3_^a^	θ_d3_^b^	λ_4_^c^	λ_5_^a^	θ_d5_^b^
Poly[(*R*)-**MAP-C**]	463	‒0.17	417	392	+0.16	292	‒0.002	282	262	+0.012
Poly[(*R*)-**MAP-C**] ann	438	‒4.65	403	387	+2.46	282	‒0.53	261	253	+0.053
copol (*R*)-75	460	‒0.10	418	391	+0.11	293	‒0.001	288	264	+0.010
copol (*rac*)	‒	‒	‒	‒	‒	‒	‒	‒	‒	‒
copol (*rac*) ann	‒	‒	‒	‒	‒	‒	‒	‒	‒	‒
copol (*S*)-75	461	+0.14	415	390	‒0.13	295	+0.008	277	261	‒0.007
Poly[(*S*)-**MAP-C**]	475	+0.16	421	393	‒0.13	291	+0.003	281	264	‒0.011
Poly[(*S*)-**MAP-C**] ann	446	+2.71	408	387	‒2.20	288	+0.18	266	259	‒0.017

^a^ Wavelength (in nm) of maximum dichroic absorption. ^b^ Ellipticity (θ_d_) normalized by the thickness and expressed in mdegree nm^−1^. ^c^ Wavelength (in nm) of the cross-over of dichroic bands.

**Table 3:** Characterization of polymeric derivatives.

Sample	Feed in mol %		*M_n_* ^a^	*M_w_*/*M_n_* ^a^	*T*_g_ (°C) ^b^
	(*R*)-MAP-C	(*S*)-MAP-C			
Poly[(*R*)-**MAP-C**]	100c	0	32,900	1.5	185
copol (*R*)-75	75c	25	33,200	1.5	190
copol (*rac*)	50c	50	33,600	1.4	192
copol (*S*)-75	25c	75	31,800	1.5	190
Poly[(*S*)-**MAP-C**]	0	100	43,900	1.4	192

^a^ Determined with SEC in THF solution at 25 °C. ^b^ Glass transition temperature determined by DSC.
